# Translation of Real-Time Infectious Disease Modeling into Routine Public Health Practice

**DOI:** 10.3201/eid2305.161720

**Published:** 2017-05

**Authors:** David J. Muscatello, Abrar A. Chughtai, Anita Heywood, Lauren M. Gardner, David J. Heslop, C. Raina MacIntyre

**Affiliations:** University of New South Wales, Sydney, New South Wales, Australia (D.J. Muscatello, A.A. Chughtai, A. Heywood, L.M. Gardner, D.J. Heslop, C.R. MacIntyre);; Arizona State University, Phoenix, Arizona, USA (C.R. MacIntyre)

**Keywords:** infectious disease medicine, epidemics, pandemics, emergencies, computer simulation, computer models, computer systems, software, disaster planning, qualitative research, bioterrorism and preparedness

## Abstract

Infectious disease dynamic modeling can support outbreak emergency responses. We conducted a workshop to canvas the needs of stakeholders in Australia for practical, real-time modeling tools for infectious disease emergencies. The workshop was attended by 29 participants who represented government, defense, general practice, and academia stakeholders. We found that modeling is underused in Australia and its potential is poorly understood by practitioners involved in epidemic responses. The development of better modeling tools is desired. Ideal modeling tools for operational use would be easy to use, clearly indicate underlying parameterization and assumptions, and assist with policy and decision making.

Modeling the population dynamics of infectious disease spread and control is a complex, multidisciplinary challenge; an increasing number of examples demonstrate its value to practical, real world problems in infectious disease management. Ongoing consultation between the modeling community, policy developers, and decision-makers is recognized as essential to maximize the working potential of modeling tools during tool development (*1*,*2*).

A dimension of modeling that has received limited recognition is rapid, real-time modeling, which provides evidence for making immediate decisions for management and control strategies in the context of an emerging epidemic. Here we use real time to mean integrating modeling into the active disease response efforts of governments and other organizations to provide timely guidance for policy development and decision-making.

A number of examples have demonstrated the value of modeling in emerging epidemics. With the recognition of the swine-origin pandemic influenza in 2009, the World Health Organization engaged with modelers in provision of evidence to help develop an informed response. Relatively simplistic rather than sophisticated simulation modeling in real time has been more commonly used for rapid policy guidance (*2*). In diverse modeling approaches, treatment capacity consistently emerged as a vital factor in the control of Ebola virus disease in West Africa in 2014–2015 (*3*–*5*). Statistical and mathematical models that provide up to 3-month ahead-of-time forecasts of disease incidence have been developed in the context of influenza and dengue (*6*–*10*). Several risk assessments of the Zika virus epidemic that emerged in Brazil in 2015 have been published (*11*–*15*).

These published examples of modeling results of large-scale, (often) international infection dynamics can assist in providing guidance for the task of overarching control strategies. However, they might not meet the more immediate, on-the-ground needs of organizations and personnel who are grappling with day-to-day management of epidemics.

Many free and commercial software tools for modeling or simulating outbreak dynamics and impact are available (*16*–*34*). Yet, studies assessing the use of modeling in public health practice are scarce. A needs assessment on the use of modeling for emergency preparedness with local and state health officials, emergency management officials, and other stakeholders was conducted for the US Centers for Disease Control and Prevention (CDC) in 2007 (*35*). The most commonly used tools were reported to be the CDC FluAid 2.0 (*18*) and FluSurge 2.0 (*19*) software, the Models of Infectious Disease Agent Study from the US National Institutes of Health (*36*), and the Bioterrorism and Epidemic Outbreak Response Model of Weill Medical College of Cornell University (*37*). 

Despite recognizing the importance of modeling, CDC study respondents were wary of basing policy decisions on modeling tools. Factors that influenced their confidence in modeling results included the credibility of the model assumptions, the integrity of the software in terms of its developers and thoroughness of its testing, the ability to scale its operation to the local level, and the consistency of results with requirements imposed by state and national jurisdictions. Other factors that influenced uptake of modeling tools included the ease of obtaining the software, the capacity of personnel to correctly use the tool, and the relevance of the modeling tool to the scenario at hand and in regular operational and epidemic/pandemic contexts. In the United States, emergency managers were more familiar with modeling than were the health sector respondents, but they did not believe health preparedness models could be used to readily inform practical action plans (*35*).

After reviewing epidemiologic modeling activities in the context of public health emergencies in the United States, Schlegelmilch et al. argued that adoption of modeling would be enhanced by establishing an interagency framework to create and strengthen relationships between modelers, operational structures, and personnel; to foster model development for decision support; and to develop the capacity of operational structures and personnel to adopt modeling for routine decision support (*38*). Akselrod et al. suggested that a synchronization matrix can be used to integrate and link key decision points in an infectious disease emergency to modeling requirements and operational aspects, such as an incident command system (*39*). They also recommended prerequisites for successfully integrating modeling into incident management operations, including training of relevant personnel, and establishing governance and procedures around use of modeling and information system development.

To better understand the limited use of modeling in the Australia outbreak emergency response context, we conducted a stakeholder workshop in April 2016 to canvas the views of policy and practice stakeholders of Australia and to document their perceived needs in relation to practical modeling tools that can be used in real time to assist in the response to infectious disease emergencies. We report here a qualitative analysis of the workshop discussion.

## Materials and Methods

To foster an informed discussion, we identified a purposive sample representing a mix of persons known to have experience or interest in mathematical modeling in the outbreak context and others who would play a leadership or management role in outbreak emergency response in Australia. Fifty-nine invitations were sent to representatives of Australia national, regional, and local jurisdictions and peak bodies involved in health protection–related activities from the following sectors: government (health protection, population health, and epidemiology), general practice, academia, and defense. Representatives were asked to suggest alternative representatives if they were unable to attend. Additional solicited and unsolicited suggestions for attendance were accepted from the initial representatives.

The workshop was facilitated by the faculty of the School of Public Health and Community Medicine of the University of New South Wales (UNSW) of Australia. Several participants elected to join the workshop by web conferencing facilities. Because of this and to facilitate maximum engagement and idea generation, the workshop was held in a large group format. The workshop started with 2 presentations by faculty: 1 introducing the purpose of the workshop with an overview of infectious disease modeling and 1 reviewing existing modeling tools. A structured discussion was facilitated by a UNSW faculty member for each of the following topics: current use of modeling tools, information requirements for epidemics and pandemics, considerations in planning a response, practical considerations in deploying modeling, and the ideal modeling tool. Workshop content and topics were agreed on by consensus among the research team. One member of the research team was allocated to facilitate and moderate discussion of each topic to prevent domination of the discussion by individual participants.

The workshop was audio recorded, professionally transcribed verbatim, and analyzed thematically. Transcripts were independently coded through repeated and close reading by D.J.M. and A.A.C., assisted by NVivo software version 11 (http://www.qsrinternational.com/product). Code lists were then cross-checked and agreed upon. A third researcher (A.H.) adjudicated over remaining disagreements.

The study was approved by UNSW Human Research Ethics Advisory panel G: Health, Medical, Community, and Social (HC16171). All participants provided informed consent. 

## Results

Twenty nine participants attended the workshop, including 6 using web-conferencing technology. National government and regional health sectors were more strongly represented than state government (Table).

**Table:** Participants at real-time modeling tool workshop, by affiliation, 2016

Affiliation	No. participants
National government including defense	8
State government	1
Regional (substate) health jurisdiction	9
Peak bodies and academia	6
Study investigation team	5
Total	29

### Current Use of Modeling Tools

Despite awareness of infectious disease modeling and a positive perception of its value, modeling, real-time or otherwise, was infrequently used in the organizations represented as indicated by the following statements: “... certainly at the moment no real-time tools [are available] that we can manipulate based on the evidence that's coming [in] as an epidemic progresses” and “... to my knowledge we don't have a lot; we don't have any communicable disease modeling expertise within the department.”

Some participants reported use of modeling for long-term planning and policy development. Most participants felt that the technical expertise needed for infectious disease modeling was not available in their organizations. “Tools can be terrific, but if there’s any lack of trust or lack of certainty on the part of the operator in what the outputs actually mean, the likelihood of success, especially in a government setting, which is a cautious setting, is low.”

### Information Requirements for Epidemics and Pandemics

The clinical, public health, and microbiology sectors are all stakeholders in the response to epidemics. Clinical stakeholders include workers in primary care/general practices, emergency departments, hospitals, intensive care services, and morgues. Their information requirements included comparing the effects of interventions, characterizing epidemics and assessing risks, planning (both short- and long-term) for health service demand, determining human and physical resource capacity, and managing logistics and work flow, as indicated by the following statements:

• “... one of the key things would be [to be] able to compare the effects of different interventions that we have available and how we could prioritize those resources to the population that are being infected.”

• “The other thing that I think is really important is some modeling around human resources. If you’re going to lose 30% of your staff, are you going to be prepared for that?”

• “Is there a distinct population that’s more susceptible?”

Understanding epidemic dynamics to inform decision-making around use of personal protective equipment (PPE), for example, was discussed: “We’re very interested in things like transmissibility from an occupational health and safety point of view... certainly during Ebola one of the big things for us was about was preparing our staff. How much PPE do we need? Who do we need to train?”

Decision support tools would be helpful for determining options and policies, and simulation of the effect those decisions have on the population and healthcare services is desirable. One participant commented that “... in a rapidly changing event, it’s expert panels... that make these kinds of decisions. I think having some modeling information rapidly would be very useful in those scenarios.”

Competing priorities are a concern when events occur simultaneously and resources have to be traded off; “... an epidemic might come at a time when other resources are also currently in play, whether it's for a heat wave or something like that. I guess being able to—I don't know if a model could do that but to—somehow look at resourcing capacity but across locations.”

### Considerations in Planning a Response

Factors in addition to published evidence that influence planning include organizational, jurisdictional, and stakeholder factors, such as communication channels, decision-making mechanisms, politics, and management hierarchies that can extend across jurisdictions and stakeholders. All sectors experience substantial uncertainty and rapid change at the beginning of an outbreak with unknown parameters, rapid changes in information, knowledge, and priorities. One participant commented that “... there was a lot of range of expert opinion on some critical issues on those expert panels. Some of that took a little while to work through and actually get a consensus as to how we would deal with that uncertainty, which is always or very often part of these things.”

### Practical Considerations in Deploying Modeling

Several participants expressed a desire to be able to use modeling tools themselves and for it to become routine. One participant stated he wanted “just a tool that is used day to day, so something that forms part of someone's workplace role and function, which they can continue on [to use] during an epidemic.... It needs to become like furniture.”

Challenges to be resolved include ensuring data, information security, and confidentiality; deciding on the platform, such as self-managed versus internal or remote service provider; educating the workforce in both tool usage and result interpretation; improving model transparency; and providing mechanisms for determining unknown parameter values. Modeling expertise needs to be present within the emergency management team. One participant stated, “I'm also nervous about using the outputs of the tool if I don't have the tool—[or] someone who really understands the tool—with me.”

### The Ideal Modeling Tool

Participants were interested in models that could predict the risk in susceptible populations; the spatiotemporal distribution of disease; the role of travel and movement on outcomes; the requirement of drugs, vaccines, and other logistics; and the effect of various interventions. As stated by a participant: “... we would like a tool that will help us plan our resources or plan our PPE needs or human resource needs....”

Geographic and temporally refined forecasts were valued by workshop attendees. Some participants stated, “... so some of the outputs about resource need from the practical point of view might be easier if they go week by week” and “... I just wanted to reiterate that the geography and how it [the pathogen]’s spreading is really crucial in terms of being able to plan.”

Ideally, modeling tools would be easy to use. One participant was interested in a tool “... that’s really straightforward with not too much training.” Tools should also be transparent about assumptions, transparent about parameterization, and provide a clear interpretation of the results. One person commented, “The most useful thing that model produced is the 3 lines at the bottom that says this means that [or] when you do this, this, and this, this will happen or we think this will happen.”

Tools would allow rapid reparameterization to adapt to local and current conditions. The trade-off between simplicity and power to provide useful information was recognized. There was a desire for tools that highlighted information gaps, “... something that you would be able to input what is known information and also to be able to see what you don't know.” 

## Discussion

Despite inviting persons who were likely to have an understanding of or experience with modeling, we found meeting participants had little practical experience in applying infectious disease modeling during ongoing epidemics. Current lack of technical expertise and confidence among the existing health protection workforce and lack of clarity about suitable modeling tools remain barriers to modeling. Nevertheless, the potential of having a modeling tool to assist with decision-making when dealing with a wide range of uncertainties commonly experienced during an epidemic was appealing to many participants. Simple and relevant modeling tools to assist in local risk assessment and resource planning were desired.

Prior use of modeling was not a prerequisite for participation in our workshop. However, neither was it in the earlier CDC modeling needs assessment (in 2007) (*35*); yet, unlike our workshop, all the CDC study respondents from the health sector reported using computer modeling tools for pandemic influenza preparedness. Similar to our study, the US respondents in the CDC study recognized challenges involved in modeling: the limited knowledge on modeling and the need to train personnel; scaling to locallevel circumstances and populations, including vulnerable populations and at-risk groups; the need to access modeling capabilities on personal computers; and the need to prepare gap analyses (*35*). The lack of modeling capacity in the public health sector continues to be recognized in the United States (*40*).

Considering infectious disease modeling as an innovative tool in public health practice, our research question concerns the adoption and diffusion of the innovation in the public health milieu. Diffusion and adoption of innovation has been studied as a predictable phenomenon (*41*,*42*). Consistent with our findings, characteristics of innovations that have been reported as antecedents of adoption include usability, utility, compatibility with existing systems, ability to be pilot tested, cost-effectiveness, reliability, and adaptability (*43*).

How should development and adoption of modeling tools proceed in the light of our findings? There is no shortage in modeling tools. However, existing modeling tools appear to have been developed largely independently from healthcare personnel and organizations and thus do not reflect the needs of these stakeholders. Academic interests might have been the driving forces for the creation of many of the modeling tools available.

User-led acquisition of modeling tools might be a solution. However, commissioning or acquiring software products, such as modeling software, is a complex and resource intensive activity and a highly risky enterprise (*44*). Formal development or acquisition of information systems that meet the needs of a business activity requires a series of interacting organizational, human resource, technical, and project management processes (*45*). Software needs to be continually updated and maintained to conform to changing operating systems, data formats, and user requirements (*40*). Critical success factors for the implementation of public sector information systems include commitment to providing authority and resources to the acquisition, a clear statement of the project mission, quality of project planning and scheduling, and availability of the necessary technology and suitably qualified technical personnel (*46*).

Another characteristic of outbreak emergencies, such as pandemics, is their relative infrequency in the day-to-day operation of health systems. Exploiting highly skilled endeavors, such as infectious disease modeling, in an emergency is challenging when the capabilities are not routinely exercised. Adopting modeling in managing more frequent, small-scale outbreaks, such as annual seasonal influenza epidemics, or incorporating modeling into emergency planning exercises might be necessary to facilitate preparedness for deploying modeling in emergencies.

Our study had some limitations. Whereas we attempted to draw participants from all states and territories of Australia and the various sectors with a stake in epidemic responses, the active participants might not have been representative of this population because of factors such as ease of travel and topic interest. On the other hand, we did offer video participation, which was chosen by some participants. Because of the workshop format, the limited number of participants from some sectors such as state government, and the consequent risk for identification of participants, we were unable to attribute comments to individual sectors. The low turnout of state government representatives possibly reflects their limited resource capacity for modeling. The study only evaluated the perceptions of the participants, which might not reflect official policy or how they would behave in practice. Some participants might have participated in the discussion more than others, potentially influencing the direction of the discussion.

Several approaches can increase understanding of the benefits and limitations of modeling and increasing its uptake in practice. First, modeling software tools should be developed to be end-user focused. They should be simple, easily understood, and provide clarity of assumptions and limitations, as well as a guide to interpretation for nonexperts. Second, training in use and interpretation of modeling needs to be available to educate the workforce in all sectors involved in outbreak emergencies. Third, modeling should be considered as an integral part of multisectoral epidemic and pandemic planning and associated governance structures. Incorporating modeling scenarios into emergency response exercises can facilitate this.

## Conclusions

Real-time modeling tools have the potential to aid public health officials make crucial decisions during public health crises. However, the tools available are poorly suited for these tasks because they were not designed with these key public health stakeholders in mind. New real-time modeling tools should be developed in collaboration with modeling experts, policy developers, and decision makers with design features that better serve the needs of the healthcare sector and end user. The tools should be simple, easily understood and provide clarity of assumptions and limitations, as well as providing a guide to interpretation for nonexperts. Real-time modeling tools that are regularly used by trained personnel and public health officials that understand and are confident in the tool’s outputs will better serve the public interest during infection disease emergencies.
